# Some Observations Connected with Measles and the Fungi of Wheat Straw

**Published:** 1862-10

**Authors:** J. H. Salisbury

**Affiliations:** American Journal Medical Sciences


*Some Observations connected with Measles and the fungi of Wheat Straw. By* J. H. SALISBURY, M. D., *American Journal Medical Sciences.*

Hon. J. Dille, of Newark, Ohio, came to my office on the evening of the 9th of December last, and stated that he was just recovering from what he believed to be an attack of measles. It was his opinion he had caught them from pitching straw from an old stack. He stated that on December 4th he pitched from an old stack a load of straw, and unloaded it in his table. Portions of the stack had become partially decayed, and was already steaming with the heat of incipient decomposition. In pitching over and picking out the best straw the air became filled with a fine dust, which he freely inhaled. The dust tasted and had the odor of old straw. This took place during the forenoon. His throat soon began to feel dry and irritated. When he returned to dinner, he could still taste and smell the old straw. This taste and smell he could not get rid of. During the following night he awoke with a very sore throat, which became much worse by morning. After getting up and dressing he was taken with a severe chill, with pains in the head and back, and felt so sick and prostrated that he was compelled to return to bed again, where he remained through the day. The chill was followed by a high fever and severe pains in the head, so much so that a portion of the time he was delirious. He felt a heavy, congested feeling about the chest, his throat and fauces were swollen and inflamed, with severe catarrhal symptoms. An eruption like that of measles appeared on his face and neck, and the *old straw* taste still continued. His fever continued high through the following (Thursday) night, with severe pains in the head.

Friday, December 6th, he felt much better, and was able to be up around the house. The fever and catarrhal symptoms had partially subsided. His eyes were sensitive, watery, and inflamed.

Saturday, December 7th, felt much better. The eruption had passed downward over the whole body, and had begun to disappear from the face. He rapidly recovered, so that on Monday, December 9th, he was moving about the streets. In the evening of the 9th he called at my office. His eyes were still red, inflamed and sensitive; throat sore, dry, and voice hoarse, and had a heavy congested feeling still about the chest. The blotches on his face could be faintly distinguished. He stated that he could still taste the old straw in his throat.

*Measles at Camp Sherman.*—At the military camp—Camp Sherman—Newark, Ohio, the measles first appeared on December 4th, the same day that Mr. Dille exposed himself to the straw dust. From November 23d to 30th, the weather was cool, damp, with considerable sleety rain and snow. During this time (there being between six and seven hundred men in camp,) many of the tents were furnished with ticks, which were filled with straw for the men to sleep on. In the centre of each tent was a fire, built in a hole in the ground, from which the smoke was led off by an underground flue, extending to the outside of the tent. The straw ticks were arranged around the fire, several in a tent, and each tick accommodated two men. On December 1st, the weather became colder and snow fell to the depth of about an inch. On the 2d—which was quite warm—this melted and wet the soil and dampened the straw ticks. December 4th, the measles made their first appearance in Camp Sherman. The men came from different parts of the county, and no one knew that he had been exposed to the disease. Some had been in camp two weeks, and no one supposed to have that disease had visited the camp. Subsequent inquiries have failed to discover any one who brought them there, or to account for their appearance from the contagion of the disease. On the first day (December 4th) there were eight cases, and within a week after there were forty. The disease then disappeared for ten or twelve days from its first appearance. Between the 14th and 16th the disease again made its appearance, and within a few days there were between forty and fifty cases more, when the disease ceased altogether. These last cases, occurring so near the usual time at which the disease ordinarily makes its appearance after exposure, renders it probable that they were communicated from the first cases.*

On the 3d of December it became warm and pleasant as growing weather in spring, and continued warm and delightful till December 10th. On the 11th and 12th it was cold and freezing. The 13th and 14th were cool. From the 15th to the 21st the weather was warm and pleasant.

*The following is the Statement of Mr. S.*—"In November, 1842, I returned home from school on a Friday. My father had the threshers, with a machine, threshing wheat. The wheat had been stowed away in the mow and in a couple of stacks outside the barn. It had undergone a slight heating, and some of the straw was mouldy. In threshing, the barn was filled with a fine dust, which tasted and smelled of old straw. I was on the straw stack all Friday afternoon and the whole of Saturday. About 4 P. M., Saturday, I became very chilly; throat and fauces became sore and swollen; a tightness and congested feeling about the chest; eyes inflamed and sensitive; and severe pains through the head and shoulders with a feeling of weariness. Following the chill, came on a high fever with increased pains and throbbing in the head and severe catarrhal symptoms. I do not remember of ever passing a more disagreeable night. The next day (Sunday) had a high fever, with severe pains in head, back and limbs; eyes swollen and sensitive, and considerable thin mucous secretion from the nose and fauces. Towards evening a few blotches made their appearance on my face. The following day (Monday) I felt rather better; the fever and catarrhal symptoms had partially subsided, and my face and neck were completely covered with blotches. My father immediately remarked that I had measles. This surprised us all, as I had not been exposed to the disease, there being none in the town where I was attending school, or in the vicinity. In the course of a couple of days, my whole body and limbs were covered with the eruption. The disease passed off like a case of ordinary measles, leaving no bad effects, save inflammation of the mucous

* For these facts I am indebted to Hon. J. Dille, and the Assistant Surgeon, Dr. James Hood.

membrane of the eyes. This I did not get rid of till the warm weather of the following spring. In from seven to fourteen days after the eruption commenced in my case, all my brothers and sisters (seven in number) were in bed with the genuine measles. My eldest brother attended school with me, and returned home when I did. These were the only cases of measles anywhere in the vicinity during that fall and winter. In my attack the disease commenced with much greater violence than in either of the others. The fever ran higher and there was greater disturbance about the head chest and throat."

Bearing upon this may be mentioned the circumstanee that in almost every instance, where our soldiers have gone into camp, in a short time after—the disease—called *camp measles*, has made its appearance, without any previous exposure—so far as known—to the measles. It should also be stated that their beds have been usually straw.

At the monthly meeting of the "*Farmer's Club*," near Newark, Ohio, last month, several of the farmers stated to Mr. Dille, that it was quite common, after threshing wheat, for persons who had been exposed much to the dust, to be taken with severe chills, followed by a high fever, catarrhal symytoms and an eruption on the face. None of them could state that any one had ever had the attack twice; nor whether they had known any cases to follow the threshing of any other kind of grain than wheat.

It is well known among swine growers, that when they bed their hogs in straw, they are affected with an eruption in the throat, fauces and roof of mouth, accompanied with coughing.

*Microscopical Examination of the Fungi of Wheat and Rye Straw.* With these observations before us, we deemed the subject one worthy of a further and more careful examination; an examination which would afford something more positive. With this view, the fungus growth of wheat straw, and the dust rising from it when disturbed, were carefully examined under the microscope. The straw used for this purpose was taken from the beds at Camp Sherman, from Mr. Dille's stable, and from stacks in the vicinity of Newark.

We then took clean, bright, wheat straw—free from fungi—packed it firmly into a box about one foot square and high, wet it with about four ounces of cold well-water, pressed on and secured the lid, and set the box near the stove in the office, where the temperature ranged from 60° to 75° Fahr. Twenty-four hours afterwards I opened the box, and found the straw in the centre of the box heated and covered with a short, white mould.

As the straw was stirred, a fine dust of spores and cells were disengaged, and rose in the air, which, when inhaled, had the odor and taste of old straw. Examined the fungi under the microscope. The plants were in all stages of development, from those just starting to those with matured sporangia.

Again the straw was moistoned, the lid secured as before, and left for forty-eight hours. The box was then opened. Found the mould had extended wider through the mass and more completely covered the straw.

We further varied the experiments in a variety of ways, and found that whenever the straw was exposed to a certain temperature, under the influence of darkness and moisture, fungi were rapidly developed. We also found that wheat or rye straw when stacked out or housed, unless unusually dry, undergoes a greater or less degree of heating, fermentation or decay, during which process a variety of fungi are developed, having the appearance of mould or mildew on the straw. When this straw is disturbed or agitated in any way the surrounding air becomes filled with innumerable spores and cells of the broken and comminuted fungi. The individual cells and spores are too minute to be distinguished by the naked eye. They can only be seen when many are together and the air filled with them; then they appear like a thin smoke. or fine dust. Suspended in the air they are freely inhaled, tasting and smelling of old straw. This taste and smell is often quite persistent, lasting for hours. The air may be filled with them though invisible to us; but generally their presence can be discovered by the taste and smell. * * * * * * *

*Inoculation of the Human System with the Spores and Cells of the Fungi of Wheat and Rye Straw.*—Case I. At 10 o'clock P. M., February 11th, 1862, I inoculated my arm with the spores and cells of the fungi of wheat straw, which I obtained by placing a straw—covered with the plants—on a plate of glass, and hitting it with a few slight taps. On removing the straw, under and both sides of it was a white, cloudy band, about one-third of an inch wide, running across the glass. These spores and cells lay so thick on the glass that, to the naked eye, they seemed to touch each other. The straw from which I obtained these cells came from a stack near this place, and was the same kind of straw as that used for beds at the camp. Under the microscope the fungi presented the same appearance, and the cells disengaged in agitating the straw were precisely similar.

Wednesday, Feb'y 12th, perfectly well. No inflammation or itching around the point of inoculation.

13th.—Slight nausea. A very slight redness and itching at inoculating point.

14th.—Got up with a feeling of lassitude and nausea, which continued all day. The redness and itching of inoculating wound increasing; had difficulty in keeping warm; chilly all day; occasional sneezing; eyes sensitive; had a peculiar feeling about the scalp, as if red pepper or mustard had been rubbed into the pores.

Saturday, Feb. 15. Nausea and lassitude continue; occasional sneezing; flashes of heat over the whole body; itching and inflammation of the wound on the arm increasing; thoughtlessly rubbed off the scab, which was about three lines in diameter. The peculiar smarting, burning, congested sensation over the whole scalp, has increased since yesterday. It extends into the bone, with pains through the forehead and temples. A few blotches have made their appearance on the face and neck. Eyes weak and inflamed, so much so that I could not use them to read over half an hour during the evening. A heavy oppressive feeling about the chest; mucous membrane of fauces and throat dry and irritated; feel as if I had a severe cold.

Sunday, Feb. 16. Had a sensation of weariness and drowsiness, with nausea, all day. Eyes red, inflamed, and sensitive; smart, so that I cannot use them to read by gaslight. Whole scalp feels sore, with a constant congested, burning sensation all through it to the bone. Arm itches; redness as large as a dime. A heavy congested feeling about the chest; have had more or less fever since Saturday morning. Throat and fauces dry and swollen, and voice hoarse. Pains in back and head have been almost constant since Friday last.

Monday, Feb. 17. The burning sensation of the scalp still continues.—Eyes weak and inflamed; cannot use them long at a time, without pain. There is still slight fever and nausea.

Tuesday, Feb. 18. Nausea; face feels as if it had been exposed to the heat of an open fire till it had become inflamed. The peculiar burning soreness of the scalp is somewhat relieved. Eyes still sensitive; catarrhal symptoms and fever less than yesterday.

Wednesday, Feb. 19. Very much better; the soreness of scalp almost entirely relieved: blotches and redness of face disappeared; catarrhal symptoms and fever gone; eyes quite well.

Case II. Wednesday evening, Feb. 19th. Inoculated myself again in the same place, with the spores and cells of fungi as before.

Thursday, Feb, 20th. Feel perfectly well, except a slight sensitiveness of the eyes.

Friday, Feb. 21. Same as yesterday.

Sunday, March 2d. Have felt perfectly well since Feb., 21st. Eyes completely recovered.

Monday, March 3d. The last inoculation has produced no effect upon the system, that I can discover.

Case III, Wednesday evening, Feb. 19, 1862, inoculated my wife on her arm, with the spores and cells of the straw fungi. The cells were taken from the same group as those used in the second inoculation of my own arm, on the same evening.

Thursday, Feb. 20. Perfectly well all day.

Friday, Feb. 21st. During the day, a dry constricting feeling of the throat made its appearance, and grew much worse during the following night. Voice hoarse; has felt chilly through the day, with a feeling of lassitude and drowsiness. Nausea; ate no dinner. Throat and fauces inflamed.

Saturday, Feb. 22d. Nausea; but little appetite; severe pains through the forehead and temples; tongue considerably furred; throat feels dry and inflamed, with a very disagreeable constricting feeling, as if it would close up. A tumid appearance of fauces; voice hoarse; slight fever.

Sunday, Feb. 23d. All through last night her throat felt as if it would close up. Rest very much disturbed. In the morning, throat felt better. Occasional sneezing; voice hoarse; some pain in swallowing. Stupid, weary, and inclined to sleep.

Monday, Feb. 24. Throat did not trouble her much last night; still hoarse; head stopped up, as if with a cold; towards evening a fullness and throbbing about the head, which felt sore.

Tuesday, Feb. 25th. Had rather a restless night; head feels sore, swollen and heavy, as with a severe cold; eyes sensitive; catarrhal symptoms severe; heaviness about the chest; slight cough; considerable lassitude and drowsiness; slept from 10 A. M., to 3 P. M.; but little appetite. Had through the day occasional sensations of deafness; slight redness in spots under the skin on the face. During the evening the pains in the head were relieved, and bowels became tender and sore.

Wednesday, Feb. 26th. Had a good night's rest; head relieved; eyes still sensitive; catarrhal symptoms subsiding; chest feels easier; bowels very sore and tender to the touch. Appetite returning; redness on arm nearly gone; slight itching yet.

Thursday, Feb. 27th. Rapidly recovering; head and eyes feel quite well; bowels still slightly tender.

Friday, July 28th, quite well.

It will be seen from this case, that although there was scarcely any perceptible blotches, yet the other symptoms, such as chills, followed by fever, pains in the head, catarrhal symptoms, nausea, lassitude, &c., were all present. The disease commenced in the head, throat and fauces, and passed downward, the bowels being very sore after the head, throat, and chest were releived.

Case IV. On Sunday, March 23d, 1862, Chas. B. Pierce, a fine healthy boy, six years of age, was exposed to measles, by contact with the disease.

March 26th, seventy-two hours after the exposure, inoculated him with the fungi of wheat straw, The fungi was grown in my office, and shaken off from the straw on plates of glass, between which the spores and cells were preserved for use. On the second day after the inoculation (March 28,) a redness appeared around the inoculating point, about the size of a dime. This was preceded and accompanied by catarrhal symptoms resembling a slight cold. Did not complain. Played out of doors every day. This redness at the point of inoculation soon disappeared; the catarrhal symptoms subsided, leaving no bad effects; an on April 2d, he was perfectly well.—Forty-two days have passed since this boy was exposed to the disease, and there are no signs of measles yet.

Case. V to IX. Mr. Bartholomew, of Newark, Ohio, has a family of seven children, ranging from three to seventeen years of age. On Wednesday morning, April 3d, Franklin Bartholomew, the next to the oldest son, broke out with measles. On Saturday evening, April 5th, three days after Franklin came down with the disease, and three days after the exposure of the entire family, I was called upon by Dr. Teller, their family physician, to go with him and inocutate the other six childred and the mother, none of whom had ever had the disease. We inoculated the mother, and four of the children, leaving two boys—one thirteen and the other seventeen years of age—without being inoculated. On April 14th, the boy seventeen years of age, and on April 16th, the one thirteen years of age broke out with the disease. It has now been five weeks since the exposure of the mother and the four children inoculated. Although there has been three successive cases of measles in the house, none of those inoculated have had any symptoms of the disease. From twenty-four to thirty-six hours after the inoculation, they all had symptoms, resembling a slight cold, with a little chilliness, catarrhal symptoms and sneezing. Beyond this they have been perfectly well from the date of the inoculation to the time of this writing, May 4th.

The inoculation does not produce a pustule and scab, like the vaccine virus, but simply a redness, around the wound, like a measle blotch.—There is seldom any soreness, but usually a simple itching sensation for two or three days, extending generally from the second or third to the fifth or sixth day after the inoculation.

CASES X to XIII. April 12th, inoculated with rye straw fungus Mrs. ——, and two of her children, none of whom had ever had measles, and who had been exposed to the congation of the disease from a case of genuine measles in the family, which broke out April 6th. On the evening of the 13th and morning of the 14th, they all had symptoms of chilliness followed by fever, catarrhal symptoms, slight cough and sneezing. The inoculating wound became red over a surface about the size of a dime, presenting the appearance of a measle blotch.

Their symptoms were so slight that the children were not kept in doors, and the mother was not prevented from attending to her ordinary duties.

On the 18th they had all quite recovered. It is now four weeks since the exposure, and no signs of measles in any of the cases inoculated.

From the inoculations so far as they have gone, in from twenty-four to seventy-two hours, the effects begin to show themselves in lassitude, chilliness, catarrhal symptoms and pains through the forehead and temples. It is highly desirable that these experiments should be extended further.—For this reason we publish thus early our observations and experiments (much more limited than we could have desired, on account of the difficulty in this place of obtaining subjects who are willing to sacrifice a few hours' health to such purposes) that others in larger places, who have greater facilities in the way of hospitals, &c., for carrying out more extended series of experiments under the eye of the attending physicians, may take up the matter and aid in its further investigation.

I have not been able to distinguish thus far any difference between the eruption and attendant symptoms of genuine measles and "camp measles," or straw measles. When the disease is communicated to the human subject, however, by inhaling the spores and cells of straw fungi, the eruption appears to follow the exposure or inhalation in from twenty-four to ninety-six hours. While in exposures to the contagion of the disease, the eruption does not usually make its appearance until from eleven to fourteen days thereafter. It is stated that inoculations made by using matter obtained from the measle blotch, or by using the tears, blood, or saliva secretions of subjects broken out with the disease, the modified type of measles which results makes its appearance generally on the sixth or seventh day after the inoculation. In inoculating, however, with the spores and cells of straw fungi, the symptoms commence usually in about twenty-four hours; though sometimes they do not make their appearance till as late as seventy-two hours thereafter.

This matter, however, requires further investigation before fully reliable statements can be made.

To what extent inoculation with straw fungi may prove effectual in protecting the human system against the contagion of measles can only be settled by careful and extended experiments.

